# Deep-sea ctenostome bryozoans: revision of the family Pachyzoidae, with description of a new genus and three new species from Zealandia

**DOI:** 10.1186/s40851-024-00226-z

**Published:** 2024-02-06

**Authors:** Thomas Schwaha, Dennis P. Gordon

**Affiliations:** 1 Department of Evolutionary Biology, University of Vienna, Schlachthausgasse 43, 1030 Vienna, Austria; 2 National Institute of Water and Atmospheric Research (NIWA), Wellington, New Zealand

**Keywords:** Deep-sea bryozoans, New Zealand, *Jeanloupia* gen. nov., *Pachyzoon grischenkoi* sp. nov., *Pachyzoon pulvinaris* sp. nov

## Abstract

Pachyzoidae is a little-known family of deep-sea ctenostome Bryozoa that until now was monospecific for *Pachyzoon atlanticum*. Originally described from the Atlantic Ocean, the genus was also found off southeastern New Caledonia in deep waters of the geological continent of Zealandia. *Pachyzoon atlanticum* forms globular to flat round colonies, living on soft, muddy to sandy bottoms with a few rhizoidal cystid appendages extending from the basal, substrate-oriented side. In this study, we investigate additional pachyzoids, collected between 1965 and 2015 from over 40 sites around New Zealand, by means of detailed morphological and histological investigations. In total, several hundred colonies were encountered in the NIWA Invertebrate Collection, comprising two new species of the genus *Pachyzoon*, *P. grischenkoi* sp. nov. and *P. pulvinaris* sp. nov., and the new genus and species *Jeanloupia zealandica* gen. et sp. nov.. The genus *Jeanloupia* is characterized by small disc-shaped colonies with highly elongated peristomes and a quadrangular aperture, distinct from the round apertures of the genus *Pachyzoon*. Pachyzoid species differ in colony structure and shape, apertural papillae and polypide features such as tentacle number or digestive-tract details. Cystid appendages are non-kenozooidal, but may originate from laterally flanking kenozooids. Based on published images, alleged *P. atlanticum* from New Caledonia is re-interpreted as *P. pulvinaris* n. sp.. Morphological characters support alcyonidioidean relationships, as previously suggested. First observations on pachyzoid reproduction show macrolecithal oocytes and brooding of embryos, which seems to be the general pattern for this family. The occurrence of three new Zealandian species in a comparatively small geographical area far from the Atlantic indicates a high possibility of more species to discovered.

## Introduction

Bryozoa is a phylum of colonial suspension feeders. Colonies are composed of iterated modules, zooids, consisting of an exterior body-wall (cystid) and internal soft tissues. Most of the latter comprises a gut and an eversible and retractable tentacle crown with associated neural and muscular tissue [1–3]. Depending on the taxonomic clade, the cystid may be cuticularized in various ways or mineralized via calcium carbonate incorporation.

Two clades can be distinguished among bryozoans: Phylactolaemata, a small group of freshwater bryozoans, and Myolaemata, which is predominantly marine [3]. Myolaemata is divided into the sister-taxa Stenolaemata and Gymnolaemata. The latter comprises the paraphyletic Ctenostomata and monophyletic Cheilostomata [4].

The deep sea includes the most widespread and least known habitats on earth, harboring many bizarre forms adapted to the challenging conditions of hundreds to thousands of meters of depth, with variable substrata and food availability. Numerous bryozoans, most prominently gymnolaemates, have been recorded from a variety of deep-sea habitats. Three ctenostome families occur almost exclusively in the deep sea and are adapted to live on soft bottoms: Aethozoidae d’Hondt, 1983 (emend. Reverter-Gil et al. 2016), Clavoporidae Soule in Osburn, 1953, and Pachyzoidae d’Hondt, 1983, the latter two from the same superfamily [see [5]. Aethozoids are bizarre single-zooid ctenostomes with appendages; they comprise four genera [6]. Clavoporids are club-shaped with a kenozooidal stalk of various morphologies and a capitulum carrying the feeding autozooids [e.g. [7, 8]; five genera have been described. Pachyzoidae is represented by the sole genus and species *Pachyzoon atlanticum* d’Hondt, 1983, which was recorded from various deep habitats of the North Atlantic Ocean [see [9] and nominally from New Caledonia [10]. Morphologically it has not been studied in detail.

Between 1965 and 2015, additional pachyzoid samples were collected by seven cruises conducted by NIWA (and its predecessor, the New Zealand Oceanographic Institute) in New Zealand waters. These yielded 366 pachyzoid colonies, comprising three new species and one new genus. Most were found off eastern South Island, with a few samples also collected in the Tasman Sea. Detailed histological analyses were carried out, allowing characterization of the new taxa and amplification of the family diagnosis with soft-body morphological features. For that purpose, material from the type species *Pachyzoon atlanticum* was analysed.

## Materials and Methods

**Fig. 1:**
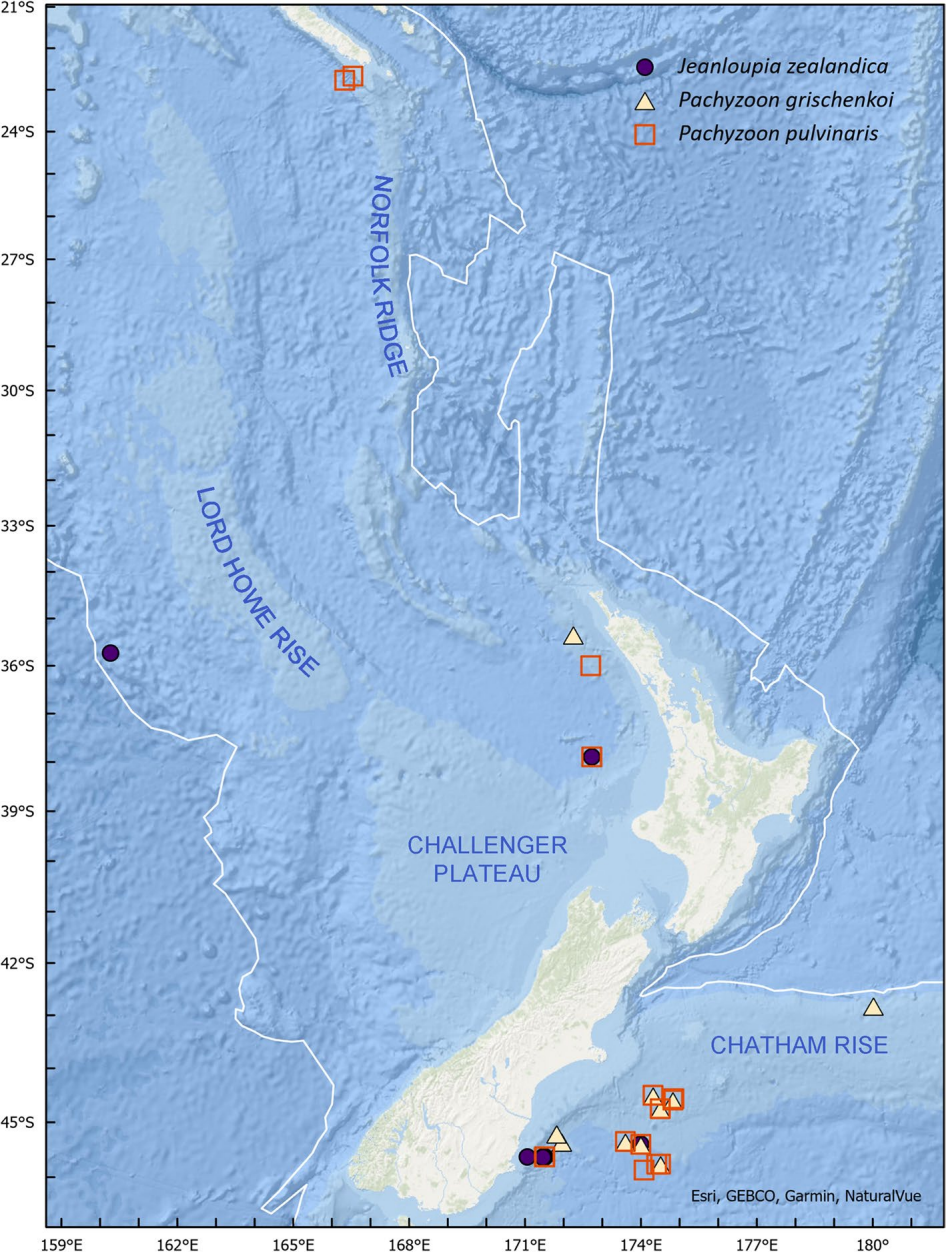
Map showing the distribution of Pachyzoidae in the Zealandian region. Latitude and longitude markers are shown at 1° intervals. The white line shows the boundary of the geological continent of Zealandia

Samples were collected by trawls, epibenthic sleds or box corers from 42 stations over a depth range of 750‒3480 m (Table 1, Fig. 1). Shipboard primary fixation was either in seawater-formalin or unknown, followed by storage in ethanol.

**Table 1:** Station data for Pachyzoidae from Zealandia

Taxon	NIWA Catalogue Number	No. of colonies	NIWA Station ID	Date	Latitude	Longitude	Depth
*Jeanloupia zealandica*	133,805	1	S154	27/10/1979	-45.4033	173.9967	1373
*Jeanloupia zealandica*	133,689	5	TAN1310/CaravelNF2	‒/‒/2013	-45.63791	171.50262	1103
*Jeanloupia zealandica*	133,691	1	TAN1310/CaravelNF3	27/08/2013	-45.63674	171.50092	1102
*Jeanloupia zealandica*	133,628	1	TAN1310/CaravelREF3	28/09/2013	-45.63661	171.46443	n.d
*Jeanloupia zealandica*	133,694	1	TAN1310/RomneyFF3	01/10/2013	-37.89405	172.72465	1552
*Jeanloupia zealandica*	133,668	1	TAN1501_C_FF1	13/01/2015	-45.642	171.50633	1126
*Jeanloupia zealandica*	133,661	1	TAN1501_C_FF4	12/01/2015	-45.63233	171.5085	1126
*Jeanloupia zealandica*	133,667	2	TAN1501_C_FF4	12/01/2015	-45.63233	171.5085	1126
*Jeanloupia zealandica*	133,663	1	TAN1501_C_FF8	12/01/2015	-45.635	171.4935	1091
*Jeanloupia zealandica*	133,656	1	TAN1501_C_MF3	12/01/2015	-45.63867	171.49717	1104
*Jeanloupia zealandica*	133,670	1	TAN1501_C_MF7	12/01/2015	-45.63417	171.50567	1116
*Jeanloupia zealandica*	133,679	1	TAN1501_C_MF8	12/01/2015	-45.63333	171.05433	1117
*Jeanloupia zealandica*	133,681	1	TAN1501_C_NF2	12/01/2015	-45.63717	171.50033	1105
*Jeanloupia zealandica*	133,662	1	TAN1501_C_NF4	12/01/2015	-45.63883	171.50283	1109
*Jeanloupia zealandica*	133,671	1	TAN1501_C_NF7	12/01/2015	-45.636	171.50267	1108
*Jeanloupia zealandica*	133,627	1	TAN1501_C_REF6	13/01/2015	-45.6415	171.46483	1024
*Jeanloupia zealandica*	133,657	2	TAN1501_C_REF6	13/01/2015	-45.6415	171.46483	1024
*Jeanloupia zealandica*	133,625	1	U202	28/09/1982	-35.7283‒ -35.7183	160.27‒ 160.2133	3480‒ 3798
*Jeanloupia zealandica*	171,002	1	TAN1501_C_FF4	12/01/2015	-45.63233	171.5085	1126
*Pachyzoon grischenkoi*	133,647	1	E416	13/10/1965	-45.35	171.95	1225
*Pachyzoon grischenkoi*	133,632	3	E417	13/10/1965	-45.2	171.8167	860
*Pachyzoon grischenkoi*	133,700	1	E881	22/03/1968	-35.3333	172.25	1371
*Pachyzoon grischenkoi*	133,698	4	F753	18/08/1966	-44.75	174.5	790
*Pachyzoon grischenkoi*	133,637	2	S138	24/10/1979	-44.59	174.82671	785
*Pachyzoon grischenkoi*	133,808	6	S147	25/10/1979	-44.5017	174.31329	760
*Pachyzoon grischenkoi*	133,636	5	S151	26/10/1979	-45.7633	174.5083	1586
*Pachyzoon grischenkoi*	133,652	25	S153	27/10/1979	-45.3517	173.5967	1386
*Pachyzoon grischenkoi*	133,811	16	S154	27/10/1979	-45.4033	173.9967	1373
*Pachyzoon grischenkoi*	85,441	3	TAN1208/57	24/06/2012	-42.81183‒ -42.8165	-179.9835‒ -179.988	972‒ 975
*Pachyzoon grischenkoi*	170,999	1	S147	25/10/1979	-44.5017	174.31329	760
*Pachyzoon grischenkoi*	171,000	1	S154	27/10/1979	-45.4033	173.9967	1373
*Pachyzoon grischenkoi*	171,000	1	S153	27/10/1979	-45.3517	173.5967	1386
*Pachyzoon pulvinaris*	133,803	1	E882	22/03/1968	-36	172.7	1217
*Pachyzoon pulvinaris*	133,624	25	F753	18/08/1966	-44.75	174.5	790
*Pachyzoon pulvinaris*	133,640	5	S138	24/10/1979	-44.59	174.82671	785
*Pachyzoon pulvinaris*	133,653	36	S140	24/10/1979	-44.565	174.8533	750
*Pachyzoon pulvinaris*	133,810	23	S147	25/10/1979	-44.5017	174.31329	760
*Pachyzoon pulvinaris*	133,649	26	S150	26/10/1979	-45.7667	174.4083	1640
*Pachyzoon pulvinaris*	133,806	112	S151	26/10/1979	-45.7633	174.5083	1586
*Pachyzoon pulvinaris*	133,699	6	S152	26/10/1979	-45.8717	174.0817	1676
*Pachyzoon pulvinaris*	133,631	21	S153	27/10/1979	-45.3517	173.5967	1386
*Pachyzoon pulvinaris*	133,802	7	S154	27/10/1979	-45.4033	173.9967	1373
*Pachyzoon pulvinaris*	133,695	1	TAN1310/CaravelFF1	27/09/2013	-45.63201	171.50234	1117
*Pachyzoon pulvinaris*	133,641	1	TAN1310/RomneyNF3D	01/10/2013	-37.89405	172.7295	1552
*Pachyzoon pulvinaris*	133,675	1	TAN1501_C_FF6	11/01/2015	-45.63217	171.51167	1123
*Pachyzoon pulvinaris*	133,674	1	TAN1501_R_NF2	08/01/2015	-37.89383	172.729	1555
*Pachyzoon pulvinaris*	133,694	2	TAN1310/RomneyFF3	01/10/2013	-37.89405	172.72465	1552
*Pachyzoon pulvinaris*	171,003	1	S151	26/10/1979	-45.7633	174.5083	1586
*Pachyzoon pulvinaris*	171,004	1	S140	24/10/1979	-44.565	174.8533	750
*Pachyzoon pulvinaris*	171,005	1	S140	24/10/1979	-44.565	174.8533	750
*Pachyzoon pulvinaris*	171,006	1	S153	27/10/1979	-45.3517	173.5967	1386
*Pachyzoon pulvinaris*^a^	—	2	BIOCAL KG 22	28/08/1985	-22.774	166.33217	2103
*Pachyzoon pulvinaris*^a^	—	1	BIOGEOCAL	07/04/1988	-22.67367	166.54533	595

^a^Data from Muséum National d’Histoire Naturelle, Paris

Specimen documentation and imaging was conducted using a Nikon SMZ25 (Nikon, Tokyo, Japan) stereomicroscope equipped with a Ds-Ri2 camera or a Hirox RH2000 microscope (Hirox, Tokyo, Japan). For histology, colonies or pieces of colonies were dehydrated in acidified dimethoxypropane followed by several rinses in pure acetone before infiltration in Agar low-viscosity resin (Agar, Stansted, UK). Cured resin blocks were serially sectioned with a Diatome HistoJumbo diamond knife (Diatome, Switzerland) at 1 µm thickness on a Leica UC6 ultramicrotome (Leica Microsystems, Wetzlar, Germany). Sections were stained with toluidine blue and sealed in resin. Analysis and documentation were conducted using a Nikon NiU microscope equipped with a Ds-Ri2 camera. Section series were converted to greyscale and enhanced in contrast with FIJI [11] before being imported into the reconstruction software Amira 2021.1 (ThermoFisher). Structures of interest were manually segmented and afterwards displayed as surface models. Surrounding tissues were displayed as volume renderings. Snapshots were taken using Amira software.

## Results

### Family Pachyzoidae d’Hondt, 1983

#### Description

Colonies free-living, discoidal or globular, usually one to several mm in size. Colonies with c. 10‒100 autozooids. Autozooids polygonal, normally tightly arranged, with orifices in close proximity on frontal side of colony. Orifices radially symmetrical or quadrangular, apertural papilla or long peristome may be present. Vestibular wall long. Autozooids with non-kenozooidal rhizoids attaching colony to substrate on basal side. Lateral kenozooids at colony margin with rhizoid processes common. Rhizoids usually non-muscular, muscles rare. Cystid cuticle wrinkled and often arborescent, commonly with attached shell or test material from foreign particles, often foraminiferans. Lophophore with 24–32 tentacles. Digestive tract short with elongated cardia, caecum usually vestigial, rarely pronounced; anus vestibular. Funicular muscle from caecum to body wall present or absent. Retractor muscles attaching at lophophoral base, foregut and cardiac portion of midgut. Parietal and apertural muscles thin and diffusely dispersed in zooids, not concentrated into regular bundles. Duplicature bands numerous on oral polypide side or totally lacking. Orificial and diaphragmatic sphincter not detected. Collar elongated, highly wrinkled, radial or quadrangular. Interzooidal pore plates simple with few special cells. Spermatogenic tissue at lateral zooidal walls, more basally. Ovaries basally associated, oocytes generally macrolecithal, internal brooding probably in tentacle sheath.

##### Genera

*Pachyzoon* d’Hondt, 1983; *Jeanloupia* gen. nov.

##### Genus

*Pachyzoon* d’Hondt, 1983.

##### Diagnosis

Pachyzoids with no or very short apertural papilla, peristome usually lacking, never highly elongated. Orifice circular, collar radial and elongated. Colony usually with dozens of zooids.

##### Remarks

We define true peristomes as elongated structures of the frontal side at the orifice. These are rigid and not retractable, whereas apertural papillae are simple, papillar rims around the orifice that show more variation in size and shape as different degrees of polypide retraction affect it. In many samples, part of the retracted vestibular wall is often protruded from zooidal orifices, which gives the impression of peristomes. The term peristome has been previously used for such tubular structures [8, 10], but in pachyzoids peristomes are present only in *Jeanloupia* gen. nov. (see below).

### *Pachyzoon atlanticum* d’Hondt, 1983 (Figs. 2,3,4,5a)

**Fig. 2:**
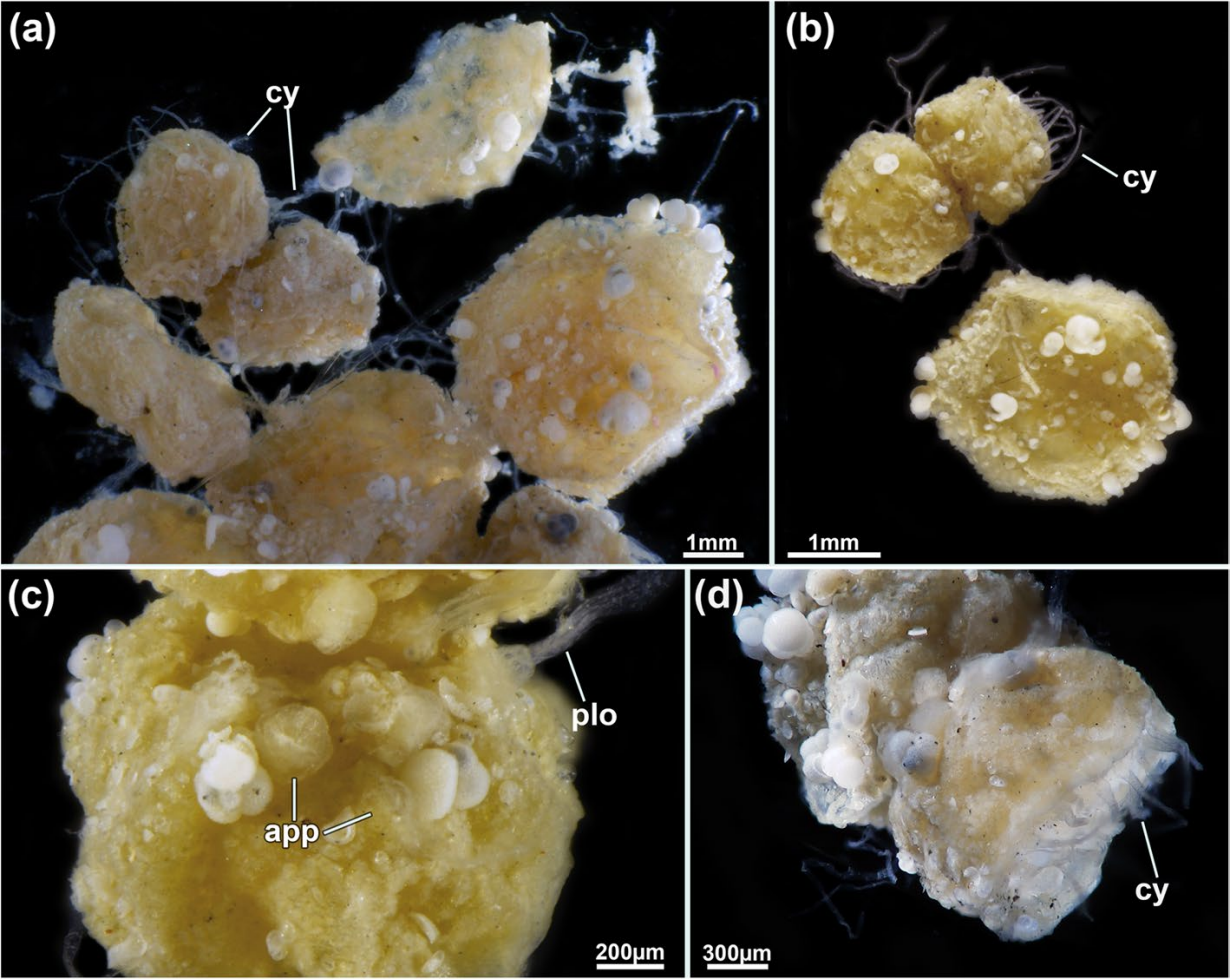
*Pachyzoon atlanticum* (**a**) General overview of several, clumped colonies. Note attached shell/foraminiferan fragments. **b** Two smaller and one larger colony, note the thread-like rhizoidal cystid appendages. **c** Detail of frontal side showing partially protruded lophophores and short apertural papillae. **d** Lateral view of colony showing rhizoids. Abbreviations: *app* apertural papilla, *cy* cystid appendage, *plo* protruded lophophore

**Fig. 3:**
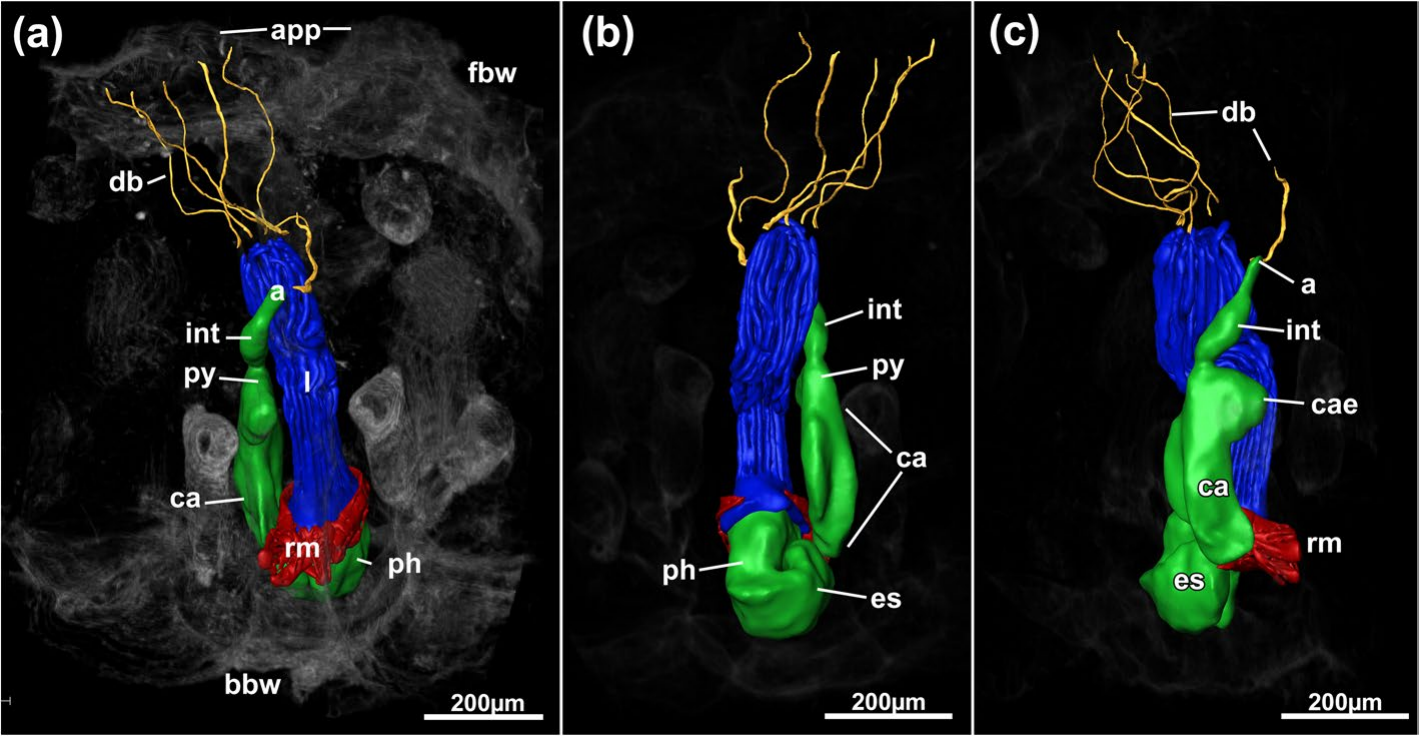
*Pachyzoon atlanticum*. 3D reconstruction based on histological sections. **a** View of polypide with surrounding areas displayed as volume rendering. **b** Lateral view of polypide. **c** Anal view of polypide. Abbreviations: a – anus, app – apertural papilla, bbw – basal body wall, ca – cardia, cae – caecum, db – duplicature band, es – esophagus, fbw – frontal body wall, int – intestine, l – lophophore, ph – pharynx, py – pylorus, rm – retractor muscle

**Fig. 4:**
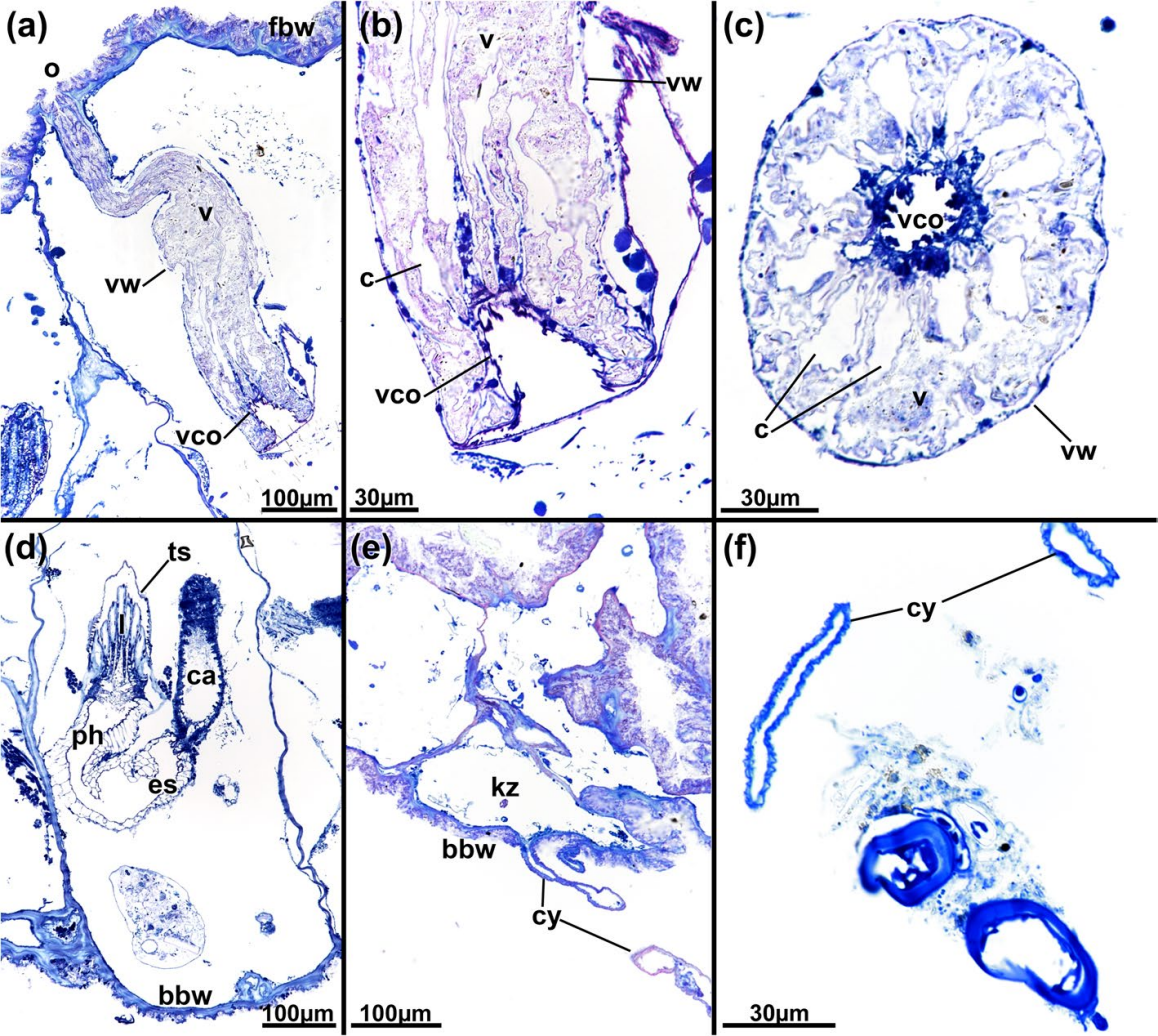
*Pachyzoon atlanticum*. Histological details. **a** Section of frontal area showing elongated vestibular wall of retracted zooid. **b** Detail of diaphragmatic area showing elongated cone-shaped diaphragm and collar folds projecting into vestibulum. **c** Cross-section of diaphragm cone and radial collar. **d** Basal part of zooid. **e** Lateral kenozooid of colony with cystid appendages. **f** Detail of cystid appendages with thin or thicker cuticle. Abbreviations: *bbw* basal body wall, *c* collar, *ca* cardia, *cy* cystid appendage, *es* esophagus, *fbw* frontal body wall, *kz* kenozooid, *l* lophophore, *o* orifice, *ph* pharynx, *ts* tentacle sheath, *v* vestibulum, *vco* vestibular cone, *vw* vestibular wall

**Fig. 5:**
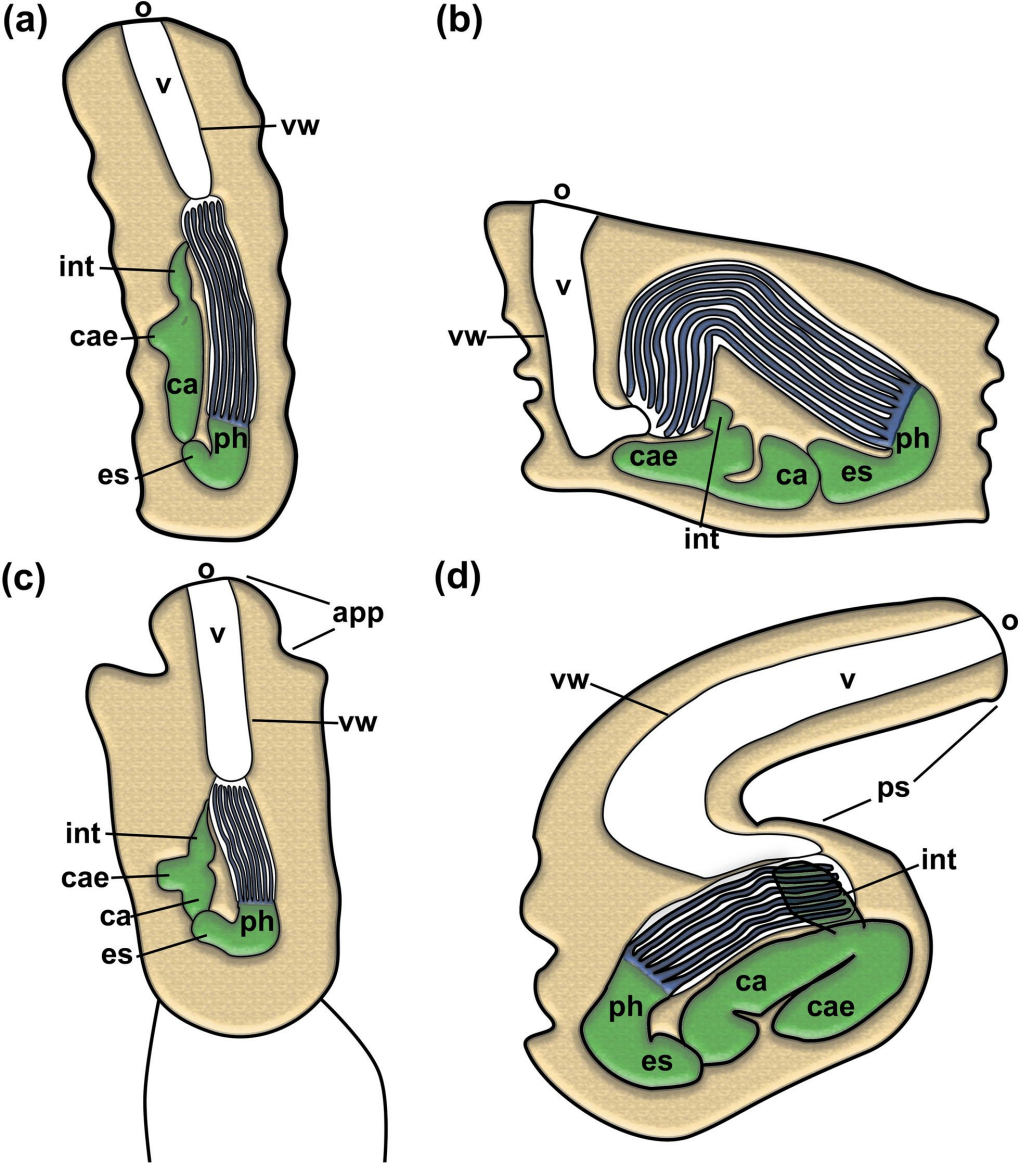
Schematic drawing of autozooids of four pachyzoid species. **a** *Pachyzoon atlanticum*. **b** *P. grischenkoi* sp. nov. **c** *P. pulvinaris* sp. nov. **d** *Jeanloupia zealandica* sp. nov. Abbreviations: *app* apertural papilla, *ca* cardia, *cae* caecum, *es* esophagus, *int* intestine, *o* orifice, *ph* pharynx, *ps* peristome, *v* vestibulum, *vw* vestibular wall

#### Material examined. NHM-UK 84.11.26.1

##### Description

Colony dome-shaped, flattened in frontobasal axis, 2.3–4.4 mm in diameter (Fig. 2). Zooids occurring as slender tubes in frontobasal axis (Figs. 3, 4d, 5a), about 1 mm long and 350 µm in diameter; cuticle complex, multilayered and sculptured with numerous surface elaborations on frontal and lateral side (Fig. 4a, e). Basal side of colony with thinner cuticle (Fig. 4d), thick and thin rhizoid cystid appendages attaching to substratum particles (Fig. 4e, f). Laterally bordered by kenozooids (Fig. 4e). Orifice on frontal side, externally inconspicuous and little pronounced (Fig. 2c, 3a). Vestibular wall extending into zooidal tube about $^{1}\!\left/ \!_{3}\right.$ of entire zooidal height in frontobasal axis (Figs. 4a, 5a), vestibulum filled with irregular flocculent material similar to ectocyst covering (Fig. 4a, b, e). Polypide with 24 tentacles; gut short with very small caecal pouch; anus highly vestibular terminating almost at diaphragm (Figs. 3, 5a). Retractor muscles originate from vertical cystid walls and insert at lophophoral base and on esophagus–cardia transition (Fig. 3). Collar epithelium large, conical with radial spikes from where large collar emerges (Fig. 4c). Spermatogenic tissue located on anal vertical zooidal wall. Multiple duplicature bands present, 5‒6 on oral and two on anal side of polypide (Fig. 3). Vestibular muscles diffuse in distofrontal area of zooid.

##### Distribution

*P. atlanticum* was first described from muddy to sandy bottoms at 800‒1600 m depth in the North Atlantic [8]. Additional records are from off Iberian coasts at similar depths [12, 13], summarized in 9].

##### Remarks

The species identified as *P. atlanticum* by d’Hondt & Gordon [10] is here considered to be *P. pulvinaris* (see below), in which case *P. atlanticum* is currently known only from the northeastern Atlantic Ocean.

## *Pachyzoon grischenkoi* sp. nov. Figures 5b, 6,7,8,9,10

**Fig. 6:**
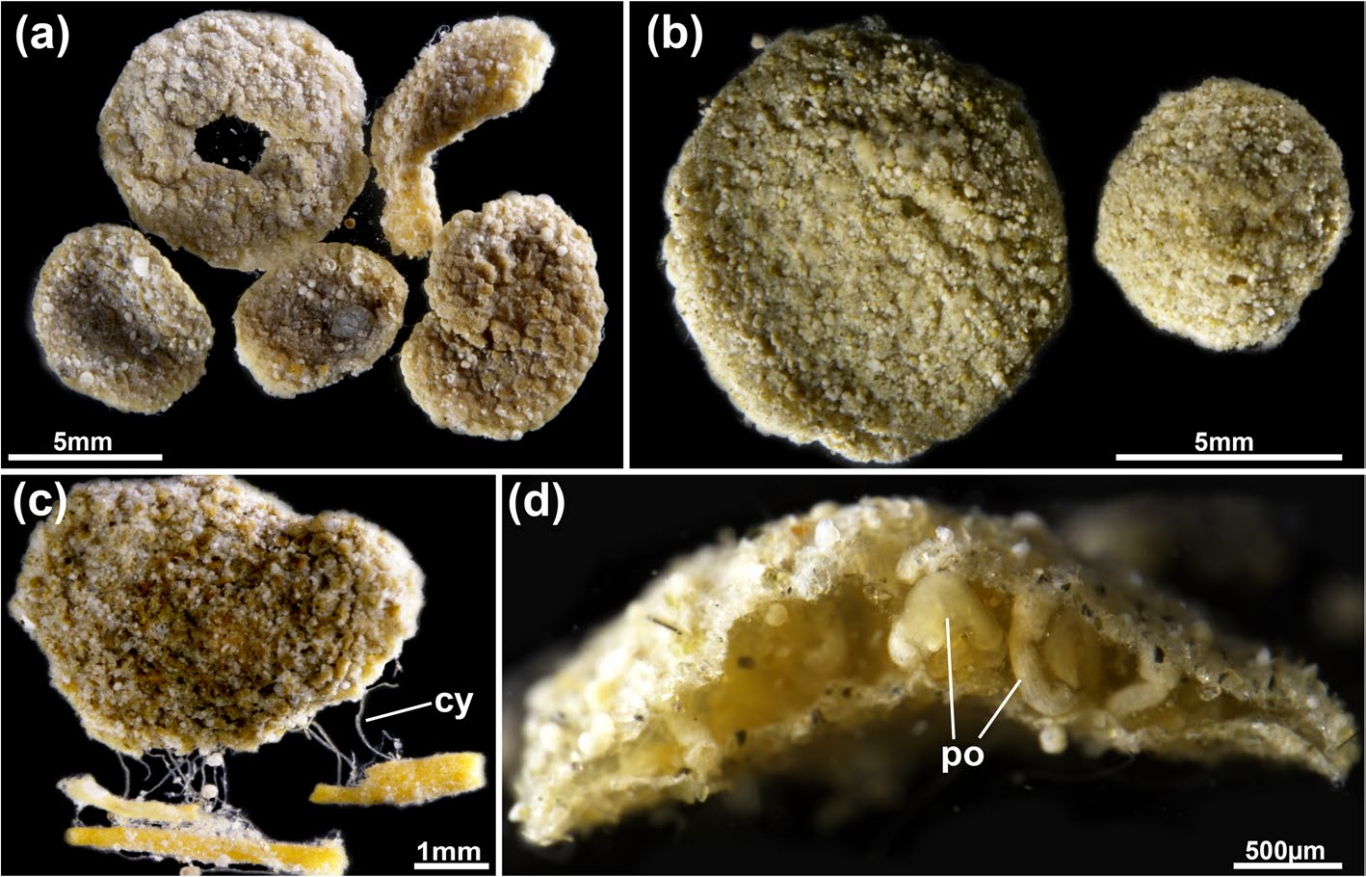
*Pachyzoon grischenkoi* sp. nov. General overview. **a** Several colonies showing size and shape range. **b** Detail of two different sized colonies. **c** Basal view of colony with cystid appendages attached to substrate (arenaceous foraminiferan tubes). **d** Laterally broken colony showing internal, single layer of polypides. Abbreviations. *cy* cystid appendage, *po* polypide

**Fig. 7:**
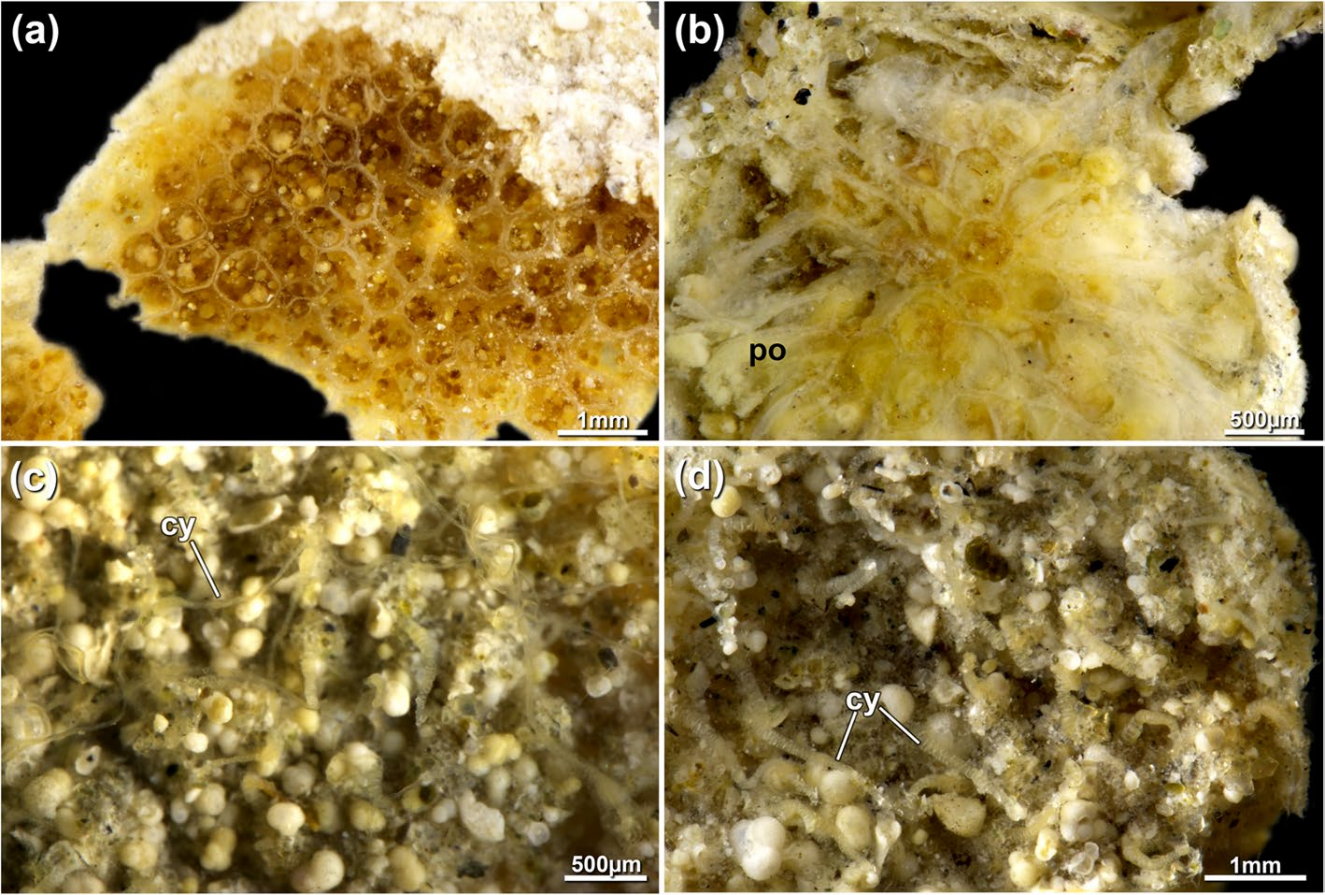
*Pachyzoon grischenkoi* sp. nov. Details of zooidal structure and cystid appendages. **a**, **b** Broken colonies showing general polygonal zooidal arrangement. Overview in (**a**), detail in (**b**). **c** Basal view of colony showing thin cystid appendages. **d** Basal view of colony showing thicker and wrinkled cystid appendages. Abbreviations: *cy* cystid appendage, *po* polypide

**Fig. 8:**
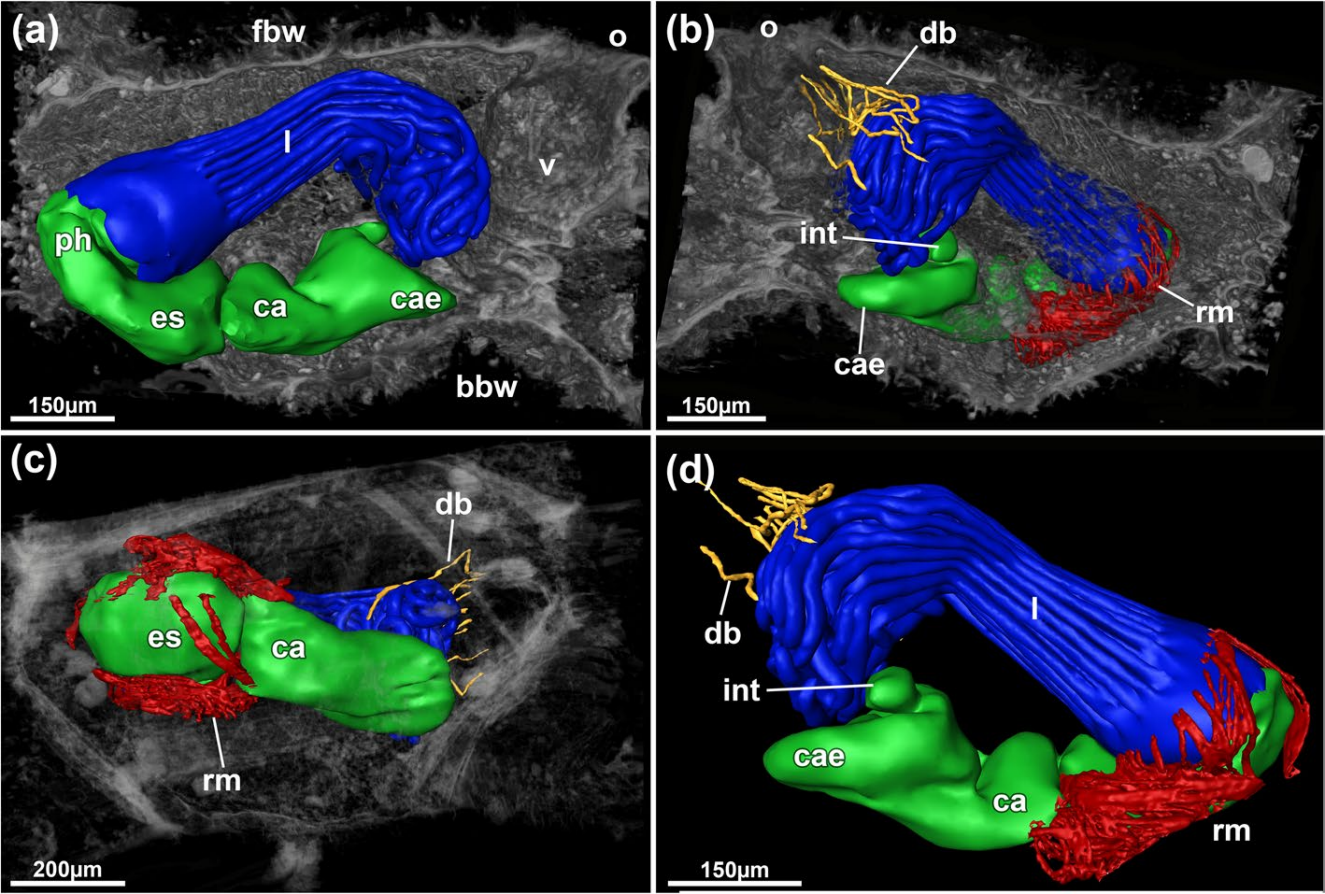
*Pachyzoon grischenkoi* sp. nov. 3D reconstruction based on histological sections. **a** Lateral view of zooid. Polypide as surface and surroundings as volume rendering. **b** Opposite view of zooid with retractor muscles and duplicature bands displayed as surface. **c** Basal view of main components of the polypide and surrounding tissues. **d** Lateral view of reconstructed polypide. Abbreviations: *bbw* basal body wall, *ca* cardia, *cae* caecum, *db* duplicature bands, *es* esophagus, *fbw* frontal body wall, *int* intestine, *l* lophophore, *o* orifice, *ph* pharynx, *rm* retractor muscles, *v* vestibulum

**Fig. 9:**
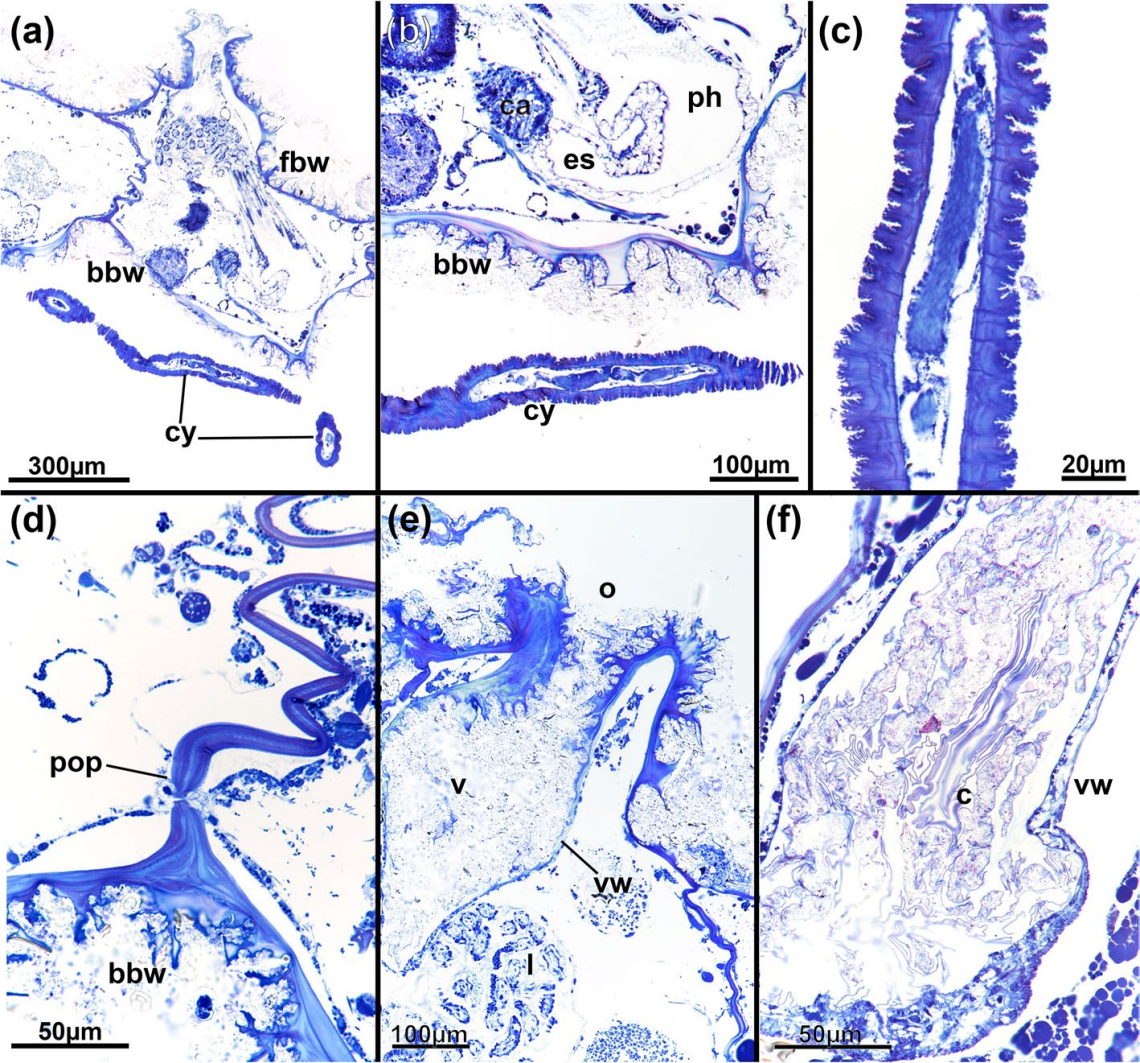
*Pachyzoon grischenkoi* sp. nov. Histological details. **a** Overview of basal area showing cystid appendages with thick cuticle. **b** Detail of single cystid appendage. **c** Detail of thick, wrinkled appendage with musculature. **d** Interzooidal pore-plate. **e** Orifice with fringed cuticle. **f** Collar within vestibulum. Abbreviations: *bbw* basal body wall, *c* collar, *ca* cardia, *cy* cystid appendage, *es* esophagus, *fbw* frontal body wall, *l* lophophore, *o* orifice, *ph* pharynx, *pop* pore-plate, *rm* retractor muscle, *v* vestibulum, *vw* vestibular wall

**Fig. 10:**
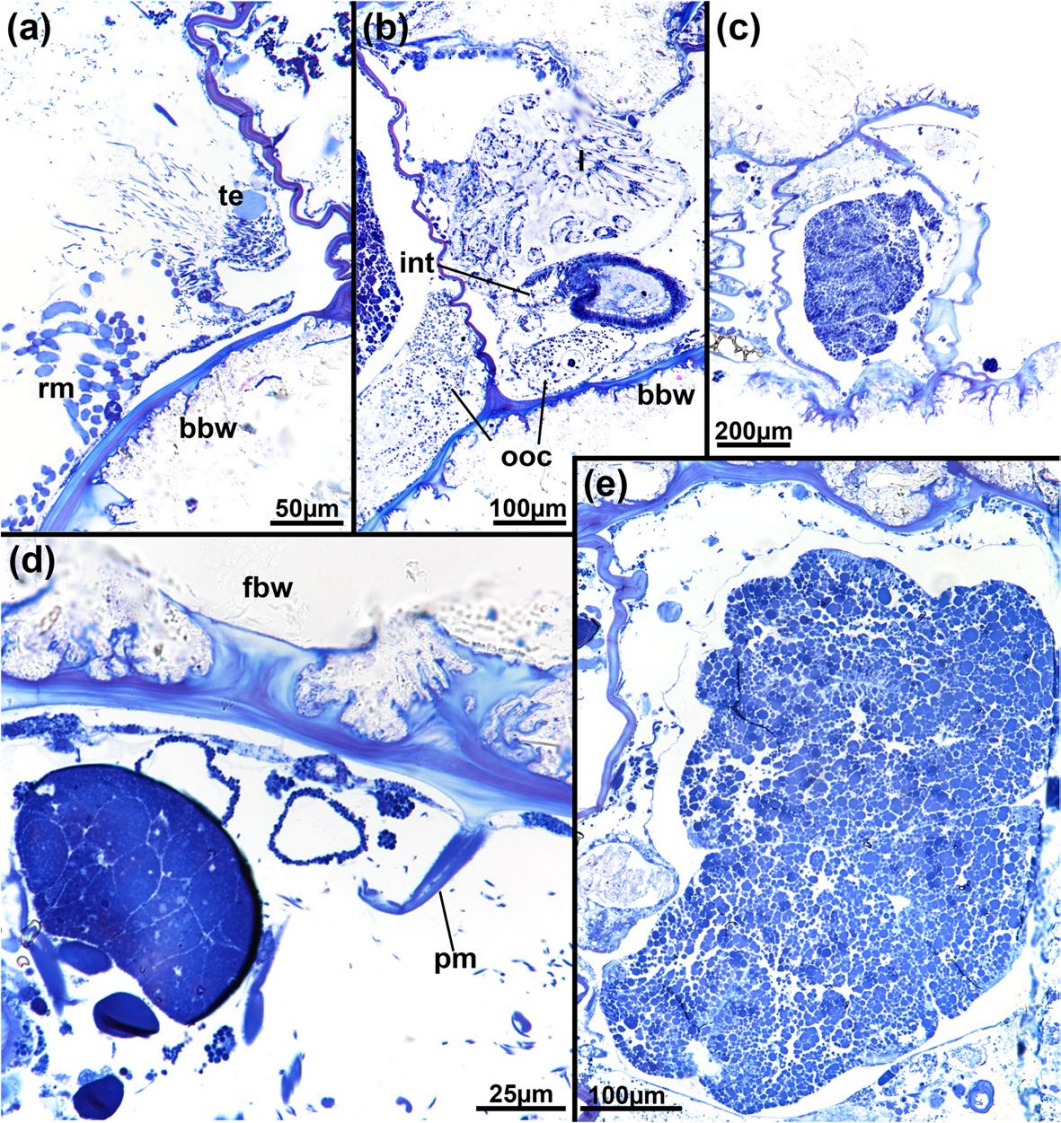
*Pachyzoon grischenkoi* sp. nov. Reproductive details. Semithin sections. **a** Basal and lateral body wall showing spermatogenic tissue on the lateral wall. **b** Basal colony area showing ovaries with oocytes of different sizes. **c** Embryo inside of maternal, degenerated zooid. **d** Early embryo. Note also cup-shaped cuticular structures. **e** Detail of embryo showing high amounts of yolk. Abbreviations: *bbw* basal body wall, *fbw* frontal body wall, *int* intestine, *l* lophophore, *ooc* oocytes, *pm* parietal muscles, *rm* retractor muscle, *te* testis

LSID urn:lsid:zoobank.org:act:02B24092-25F1-4A4F-ABC8-E2A1089C98A5.

### Material examined

NIWA 85441, 133632, 133636, 133637, 133647, 133652, 133698, 133700, 133808, 133811, 170999, 171000, 171001, totalling 69 colonies (data in Table 1).

### Type material

Holotype: NIWA 133647, paratype 1: NIWA 170999, paratype 2: NIWA 171000, paratype 3: NIWA 171001.

### Etymology

Honorific for bryozoologist Andrei V. Grischenko, who has contributed significantly to knowledge of deep-sea bryozoans including ctenostomes.

### Description

Colony flattened, mostly circular, often discoidal or oval sometimes elongated in one direction. Size 3‒10 mm diameter and c. 500‒600 µm height. Number of zooids observed approximately 50‒100 per colony. Zooids polygonal, 550–750 µm long and 410–650 µm wide, forming a single layer (Figs. 5b, 6d, 7a, b, 8), numerous sediment particles attached (Figs. 6, 7). Cuticle with strong wrinkles, often arborescent as thin extensions (Figs. 9a, b, d, e, 10d). Elongated cystid appendages occurring basally as rhizoids (Figs. 6c, 7c, d, 9a–c), these thin or thick, with thick wrinkled cuticle, muscular (Fig. 9b, c). Orifices inconspicuous and often obscured by attached particles. Vestibular wall short, extending from frontal side almost to basal side of colony (Figs. 5b, 8a). Retracted polypide longitudinal axis parallel to frontobasal axis (Figs. 6b, 8a–c). Lophophore with 32 tentacles, digestive tract with elongated cardia, small caecal pouch, very short intestine with vestibular anus (Figs. 5b, 8). Retractor muscle inserting at foregut and cardiac portion (Fig. 8c, d), multiple duplicature bands (c. 8‒10) extending on frontal tentacle sheath (oral polypide side) towards frontal body wall. Ovary with large macrolecithal oocytes on basal side, embryos brooded, probably in tentacle sheath of degenerating zooid (Fig. 10).

### Distribution

Southwest Pacific Ocean; most samples collected southeast of South Island, New Zealand, one sample from the Tasman Sea off northeastern North Island; 760‒1586 m.

### Remarks

*Pachyzoon grischenkoi* sp, nov. occurs principally in or on the surface layer of terrigenous-foraminiferal ooze. Its flattened disc-shaped colony bears some resemblance to the free-living Arctic ctenostome *Alcyonidium disciforme* [14]. Whereas colonies of the latter always develop a central hole once reaching a certain colony size, such a hole is generally missing in *P. grischenkoi*. Despite the superficial similarities of both species, clear differences are found in the cuticle, which is branching/arborescent in *P. grischenkoi* and smooth in *A. disciforme*. Also, multiple duplicature bands on the oral polypide side are only found in this species and *P. atlanticum* whereas in *Alcyonidium* four regular bands are usual [15, 16]. The more flattened colony shape, differences in tentacle numbers (24 in *P. atlanticum* and 32 in *P. grischenkoi*) and different gut structure, particularly the very small intestine of *P. grischenkoi*, clearly distinguishes *P. grischenkoi* sp. nov. from *P. atlanticum*.

## *Pachyzoon pulvinaris* sp. nov.

### *Pachyzoon atlanticum*: d’Hondt & Gordon 1996, p. 62, Fig. 2C, D. Figures 5c,11,12,13

**Fig. 11:**
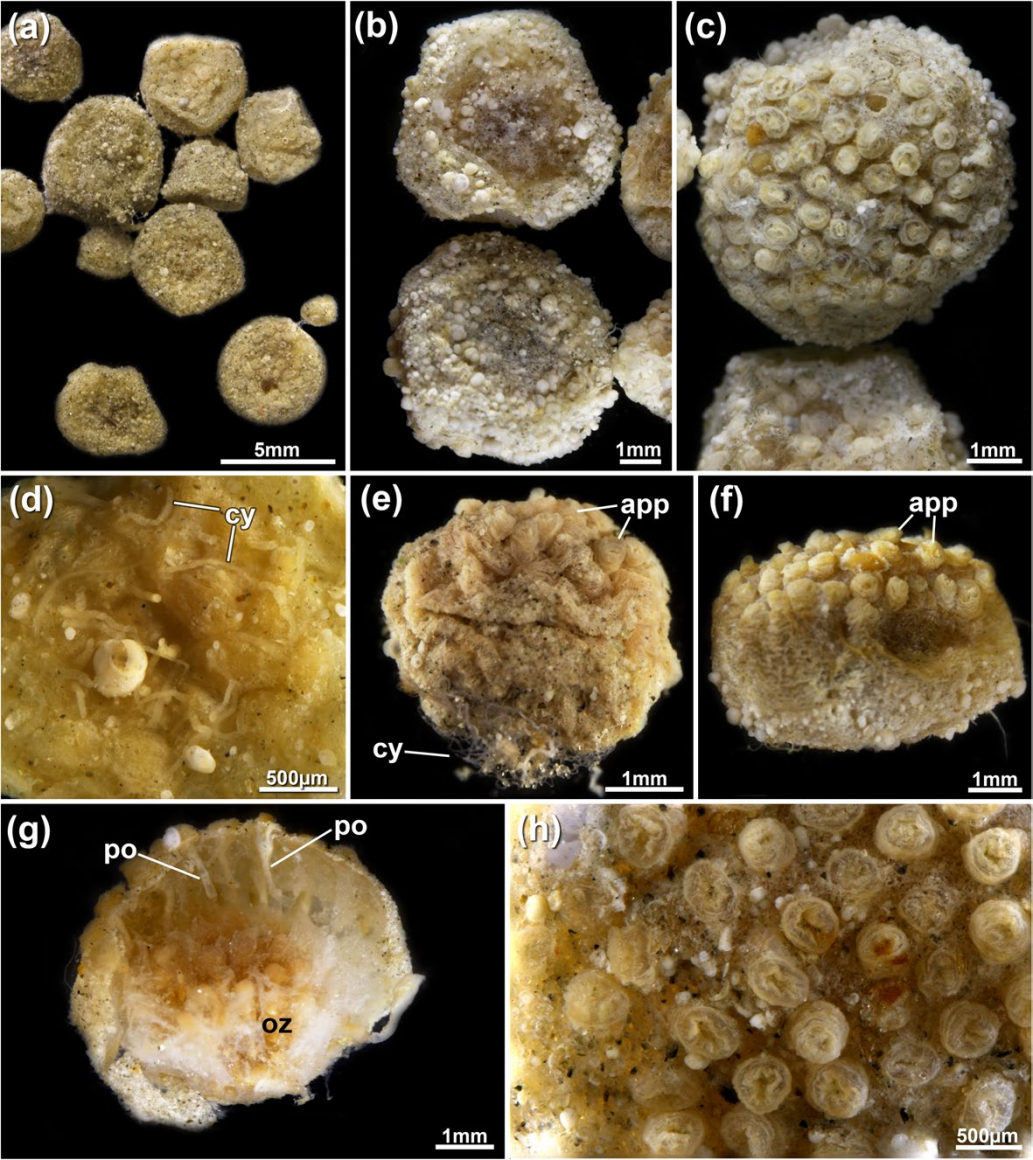
*Pachyzoon pulvinaris* sp. nov. General overview. **a** Multiple colonies. **b** Two different colonies, viewed from basal side (upper) and frontal side (lower). **c** Detail of frontal side of a colony showing multiple apertural papillae. **d** Close-up of basal side showing cystid appendages. **e**, **f** Lateral view of two colonies with basal side with more cystid appendages and less-prominent apertural papillae (**e**) or opposite (**f**). **g** Broken colony showing frontal area with functional polypides and basal area without. **h** Detail of apertural papillae on frontal side. Abbreviations: *app* apertural papilla, *cy* cystid appendage, *oz* old zooid, *po* polypide

**Fig. 12:**
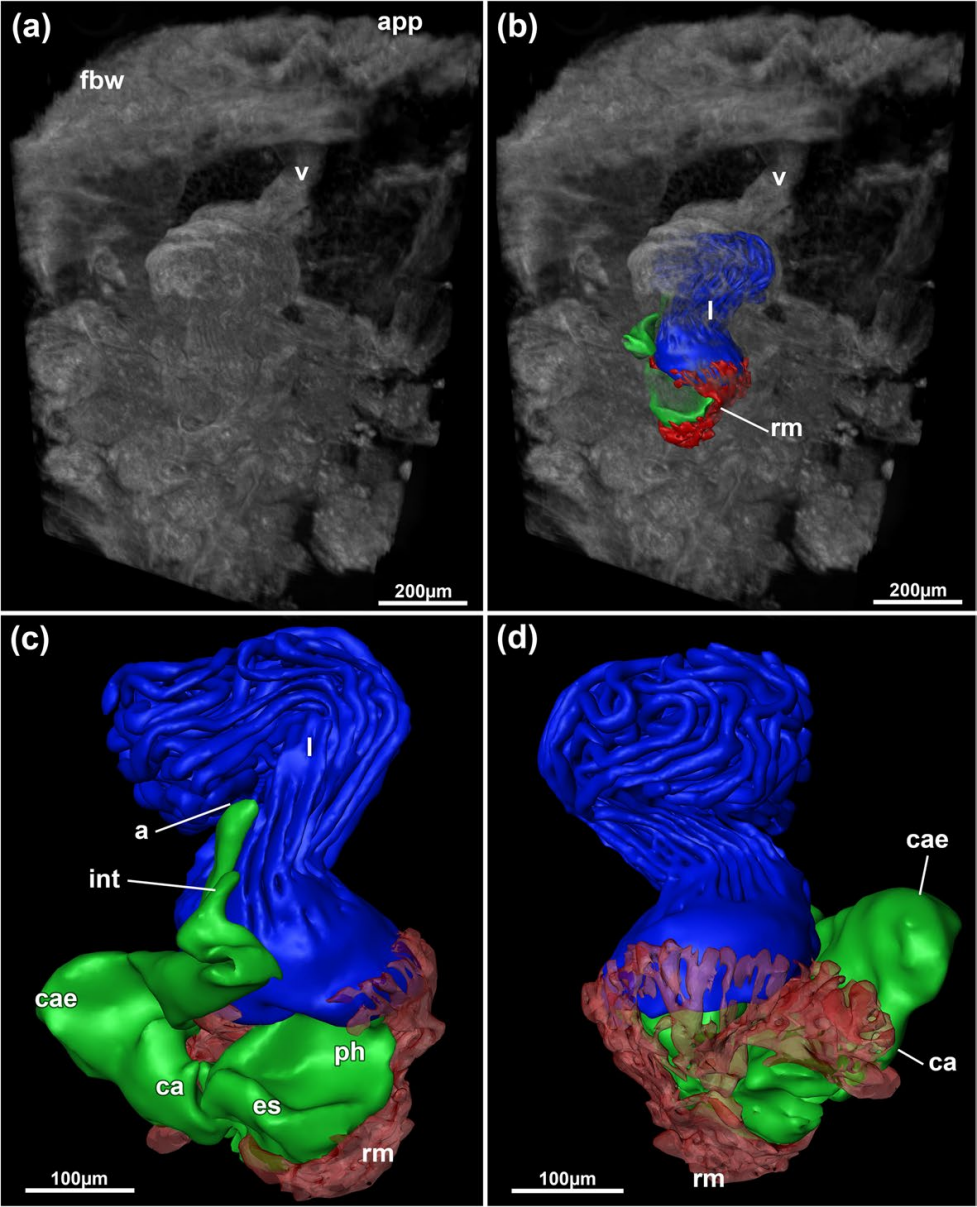
*Pachyzoon pulvinaris* sp. nov., 3D reconstruction based on histological sections. **a** Volume rendering of frontal colony area. **b** Same as (**a**) but with reconstructed polypide features displayed. **c** Close-up of polypide. **d** Same as (**c**) but from opposite side. Abbreviations: *a* anus, *app* apertural papilla, *ca* cardia, *cae* caecum, *es* esophagus, *fbw* frontal body wall, *int* intestine, *l* lophophore, *ph* pharynx, *rm* retractor muscles, *v* vestibulum

LSID urn:lsid:zoobank.org:act:90C102A2-6A74-4243-926A-9E49BB39C3A2.

#### Material examined

NIWA 133624, 133631, 133640, 133641, 133649, 133653, 133674, 133675, 133694, 133695, 133699, 133802, 133803, 133806, 133810, 171003, 171004, 171005, 171006, totalling 273 colonies (data in Table 1).

#### Etymology

Latin *pulvinaris*, cushion-like, alluding to the cushion- or sac-like form of the colony, resulting from several astogenetic zooidal layers.

#### Type material

Holotype: NIWA 171003, paratype 1: NIWA 171004, paratype 2: NIWA 171005, paratype 3: NIWA 171006.

#### Description

**Fig. 13:**
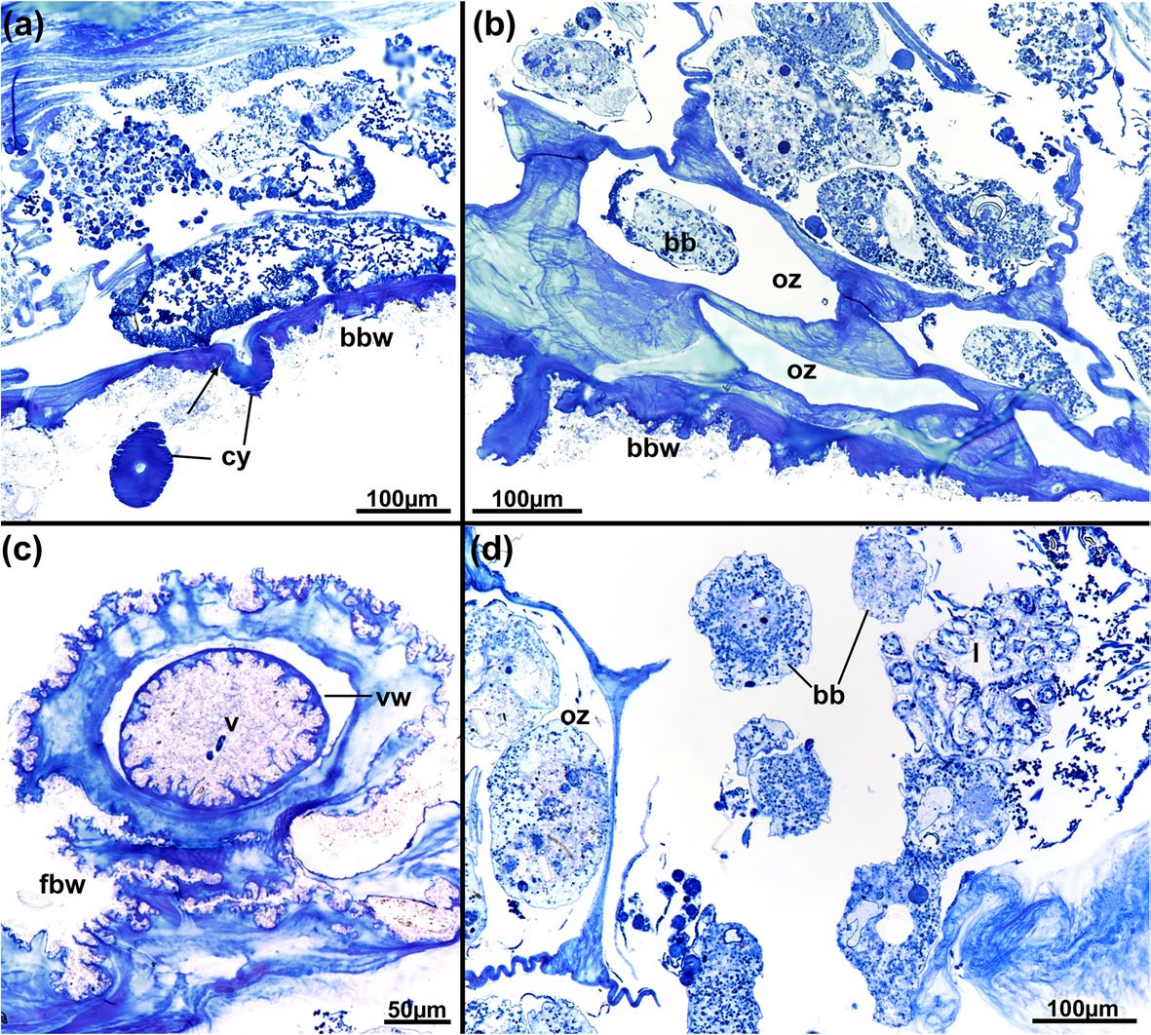
*Pachyzoon pulvinaris* sp. nov. Histological details. **a** Basal colony side with non-kenozooidal cystid appendages, unrestricted in contact with the main body cavity (arrow). Note the thick cuticle of the appendages following the remaining cuticle. **b** Basal colony area showing multiple layers of zooidal generations from basal to frontal, with degenerated polypides. **c** Apertural papilla with cross-section of vestibular wall. **d** Zooid with several large oocytes. Abbreviations: *bb* brown body, *bbw* basal body wall, *cy* cystid appendage, *fbw* frontal body wall, *l* lophophore, *oz* old zooid, *v* vestibulum, *vw* vestibular wall

Colony irregularly subspherical (Fig. 11), measuring 1.6–6.3 mm diameter, multilayered, with functional zooids on frontal side (Figs. 11g, 12a, b) and old, degenerated zooids lacking polypides towards basal side (Figs. 11g, 13b). Frontal side with regular, dense apertural papillae of individual zooids (Figs. 11c, e, f, h, 13c); zooids number around 40–50 in most analysed specimens. Thin rhizoid-like cystid appendages present on basal side at degenerated zooids, with thick cuticle, few wrinkles. No internal musculature. Encrusting particles common on basal and lateral sides, and frontally between apertural papillae (Fig. 11). Vestibular wall elongated, with deeply immerged retracted polypide along frontobasal axis of colony (Figs. 5c, 12). Lophophore with 32 tentacles, digestive tract with short foregut and cardia, caecum vestigial, slender elongated intestine (Fig. 12c, d). Retractor muscles inserting at foregut and cardiac portion of gut (Fig. 12c, d). Duplicature bands not present.

#### Distribution

Southwest Pacific Ocean; most samples collected off southeastern South Island, New Zealand, some samples from the Tasman Sea west of North Island. Also, off southwestern New Caledonia [10]; the depth range off New Caledonia (595‒2103 m) encompasses that for New Zealand samples (750‒1676 m).

#### Remarks

*Pachyzoon pulvinaris* sp. nov. can occur in high numbers in each sample; 113 colonies were collected from one station in the Bounty Trough. Colonies often show deformations owing to fixation can make measurements difficult; reliable metric data depend on the least-distorted specimens. Size measurements are thus difficult to compare and individual zooid sizes could not be determined. However, the typical cushion-shape of colonies and polypide features clearly distinguish this species from other congeners. Nominal *Pachyzoon atlanticum* described from New Caledonia by Gordon & d’Hondt [10] is here considered to be *P. pulvinaris*, as overall colony size and shape conform more to this species than to *P. atlanticum*. The little information provided shows rather globular colonies more similar to *P. pulvinaris* sp. nov. rather than the more flattened ones of *P. atlanticum*. Additional polypide features such as the general gut structure and different tentacle number (24 in *P. atlanticum*, 32 in *P. pulvinaris* sp. nov.) could support this, but data for the New Caledonian species is missing. Partially protruded vestibular walls, interpreted as peristomes as found in the New Caledonian samples, were also detected in some colonies in the current study. However, the frequency of such characters is relatively rare based on our analysis of over 100 colonies.

#### Genus *Jeanloupia* gen. nov.

LSID urn:lsid:zoobank.org:act:6EB41330-1505-4E5C-BFC2-CF0FD302F346.

##### Type species

*Jeanloupia zealandica* sp. nov.

##### Material examined

NIWA 133625, 133627, 133628, 133656, 133657, 133661, 133662, 133663, 133667, 133668, 133670, 133671, 133679, 133681, 133689, 133691, 133694, 133805, 171002, totalling 24 colonies (data in Table 1).

##### Diagnosis

Pachyzoids having straight, highly elongated peristomes with much cuticular wrinkling. Colonies typically with 11 zooids or lower. Aperture and collar quadrangular. Caecum large.

##### Etymology

Honorific for Jean-Loup d’Hondt, who first discovered and described pachyzoids.

### *Jeanloupia zealandica* sp. nov. Figures 14,15,16

**Fig. 14:**
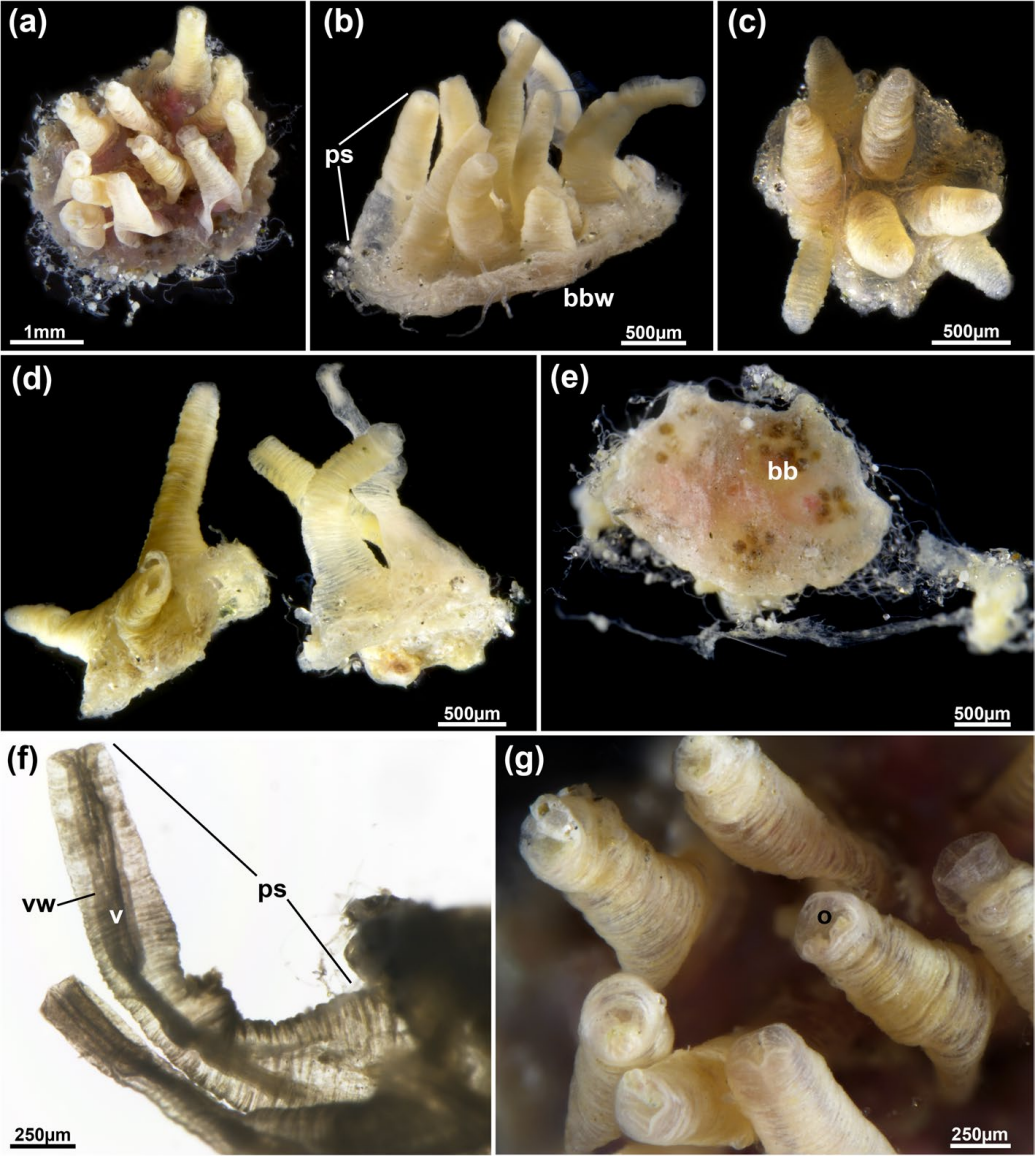
*Jeanloupia zealandica* gen. et sp. nov. General overview. **a** Colony from frontal view. **b** Lateral view of colony. **c** Colony with seven zooids, frontal view. **d** Lateral view of two small colonies of three zooids each. **e** Basally broken colony showing zooidal borders and brown bodies. **f** Lateral view of peristome showing wrinkled surface. **g** Detail of frontal apertures showing rectangular orifices. Abbreviations: *bb* brown bodies, *bbw* basal body wall, *o* orifice, *ps* peristome, *v* vestibulum, *vw* vestibular wall

LSID urn:lsid:zoobank.org:act:07D1F63A-8803-4D7B-900A-C5153BCA91B1.

#### Material examined

NIWA 133625, 133,627, 133,628, 133,656, 133,657, 133,661, 133,662, 133,663, 133,667, 133,668, 133,670, 133,671, 133,679, 133,681, 133,689, 133,691, 133,694, 133,805, 171,002, totalling 24 colonies (data in Table 1).

#### Type material

Holotype: NIWA 133628, paratype 1: NIWA 133656, paratype 2: NIWA 133668, paratype 3: NIWA 171002.

#### Etymology

Alluding to its occurrence in the geological continent of Zealandia, which includes New Caledonia and New Zealand and the adjacent seafloor.

#### Description

**Fig. 15:**
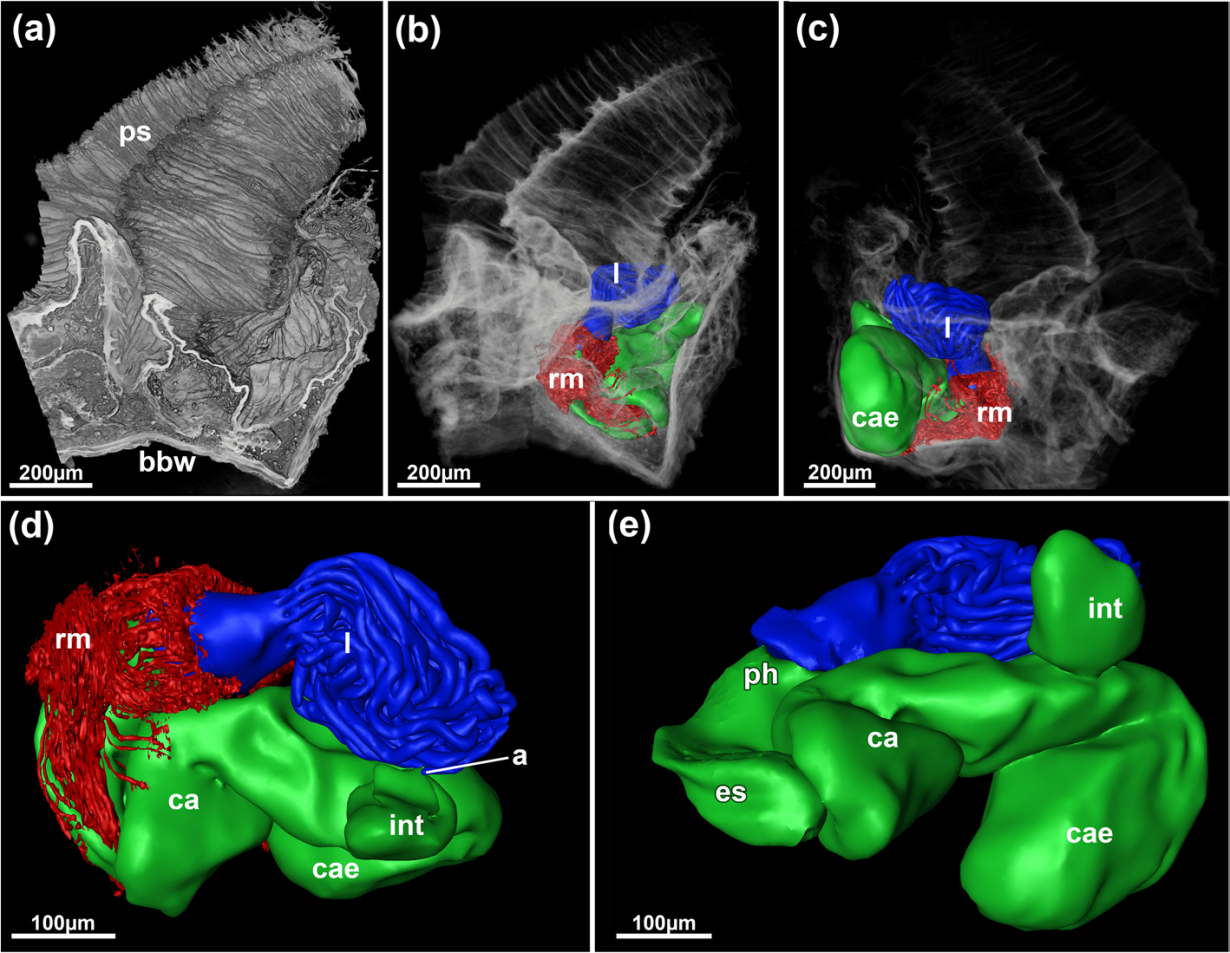
*Jeanloupia zealandica* gen. et sp. nov. 3D reconstruction based on histological sections. Volume rendering showing elongated wrinkled peristomes on the frontal side of colony. **b** Same view as (**a**), but with volume displayed transparently and polypide details shows as surface models. **c** Opposite view of (**a**) and (**b**) with more transparent volume rendering. **d** Frontal view of the polypide. **e** Basal view of polypide. Abbreviations: *a* anus, *bbw* basal body wall, *ca* cardia, *cae* caecum, *es* esophagus, *int* intestine, *l* lophophore, *ph* pharynx, *ps* peristome, *rm* retractor muscles

**Fig. 16:**
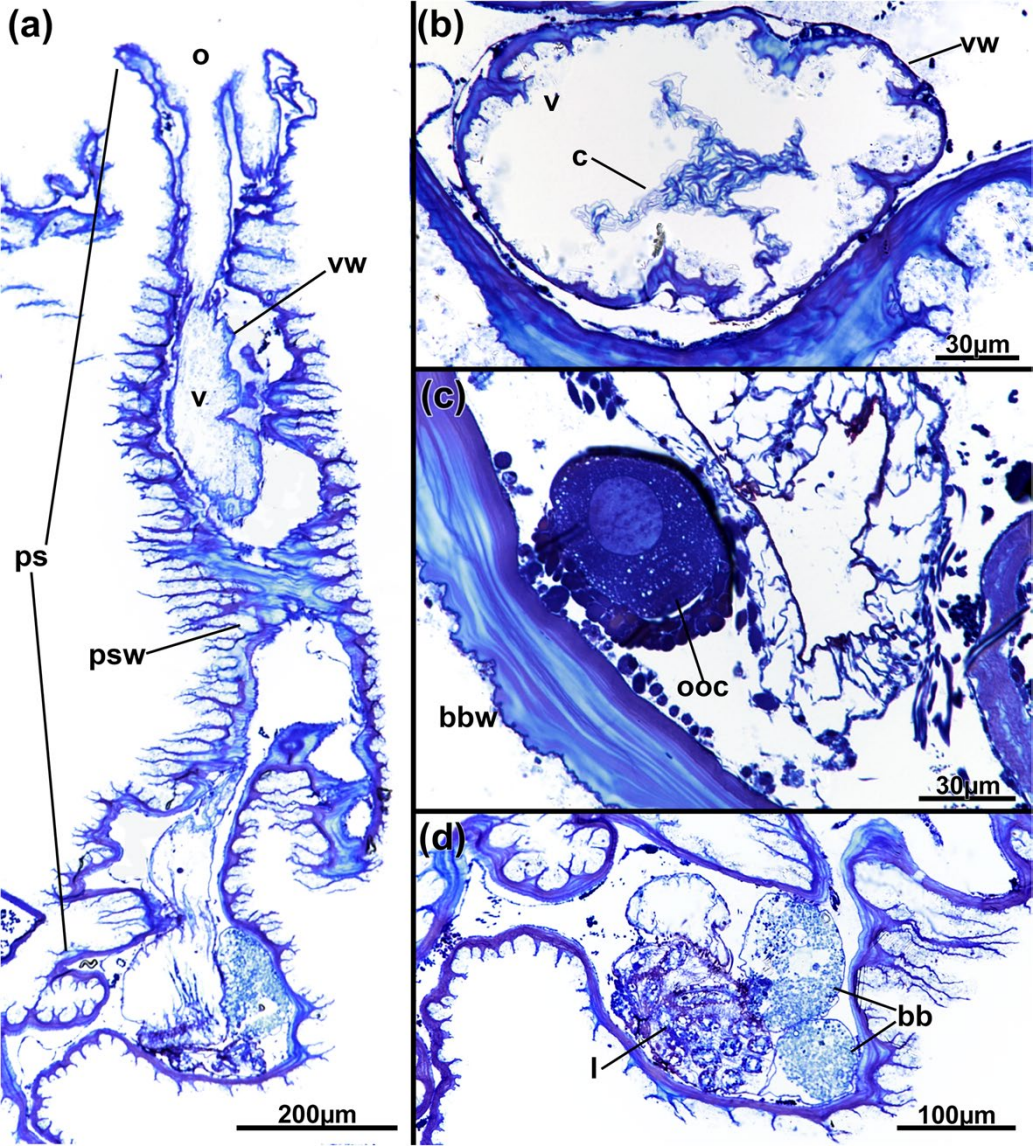
*Jeanloupia zealandica* gen. et sp. nov. Histological details. **a** Longitudinal section of peristome. **b** Cross-section of basal area of peristome showing the collar. **c** Ovary with vitellogenic oocyte surrounded by follicle cells. **d** Oocytes in basal zooidal area. Abbreviations: *bbw* basal body wall, *c* collar, *l* lophophore, *o* orifice, *ooc* oocyte, *ps* peristome, *psw* peristomial wall, *v* vestibulum, *vw* vestibular wall

Colonies small, mostly circular, 1.2‒3.2 mm diameter, comprising 3‒11 zooids (Fig. 14). Colony flattened with thickened lateral rim; very prominent elongated peristomial tubes on frontal side, these commonly bent, sometimes straight (Fig. 14, 15a-c), 1205‒2006 µm long. Basal zooidal part 890–930 µm long and 745–755 µm wide. Cuticle thick and multilayered, less conspicuous on basal side; cuticle on peristomial tubes with prominent circular wrinkles showing dendritic branching (Fig. 16a). Cuticle sometimes covered externally by attached flocculent material. Vestibular wall extending entire length of peristomial tube, with quadrangular orifice at frontal end. Collar quadrangular at diaphragm, basal end of vestibular wall (Fig. 16b). Retracted polypide restricted to flattened basal portion of zooid, not present in peristomial tube (Fig. 5d, 15b, c). Polypide with 28 tentacles. Gut with elongated cardia, large caecum present (Fig. 16d, e). Duplicature bands absent. Ovary with macrolecithal oocytes located at basal portion of zooid (Fig. 16c, d).

#### Distribution

Southwest Pacific Ocean; most samples collected off southeastern South Island, with one sample west of North Island and another west of Lord Howe Rise near the western continental margin of Zealandia in the mid-Tasman Sea; 1024‒3798 m.

#### Remarks

Specimen NIWA 133805 has unusually short peristomes that look more like apertural papillae.

## Discussion

### General structure and diagnostic characters of pachyzoid ctenostomes

Sequence data are so far lacking for any pachyzoid, and indeed for any deep-sea ctenostome (e.g. clavoporids, aethozoids). Consequently, achieving a phylogenetic understanding of deep-sea ctenostomes is challenging. The present analysis clearly shows numerous obvious differences between the four pachyzoid species, but their relatedness to each other and to other ctenostomes remains enigmatic. In agreement with previous assessments [8, 17], we can conclude from our analysis that there are several morphological characters that support a closer relationship of pachyzoids to alcyonidioidean ctenostomes. This particularly relates to: 1) a dense zooidal arrangement of polygonal zooids; 2) diffuse and spread-out parietal and apertural musculature; 3) high tentacle number as in other alcyonidioideans [5]; 4) gut morphology with a particularly short or vestigial caecum, lack of cardiac prominence and vestibular anus [18]; and 5) at least for the genus *Pachyzoon*, a circular orifice.

Individual zooid sizes are usually difficult to observe externally as their boundaries are not clear unless the obscuring covering is partially removed or colonies are damaged to show internal structures. For *P. pulvinaris *even, no zooid sizes were determined as zooidal wall were often broken und in dissected colonies also untraceable. We therefore consider the general colony shape and details in polypide features more reliable characters for species distinction in pachyzoids.

The quadrangular apertural shape in *Jeanloupia zealandica* begs the question of its affinity to pachyzoids, since apertural shapes are usually diagnostic at family level [5]. Apertures in Alcyonidioidea are circular (Alcyonidiidae, Clavoporidae), quadrangular (e.g. Pherusellidae) or bilateral (Flustrellidridae). Hence it remains ambiguous whether pachyzoid colony morphology and shape evolved independently in the genera *Pachyzoon* and *Jeanloupia*.

Duplicature bands are a common feature of all bryozoans with some victorellid and many vesicularioidean ctenostomes showing reductions [15]. Usually there are four bands, two on the anal side and two on the oral side of the polypide. Some ctenostomes have a few additional bands [19, 20], and some cheilostomes also show four on each side [3], but multiple oral-sided duplicature bands as are found in *P. grischenkoi* sp. nov. and *P: atlanticum* have not been described. Their function and significance remain unknown.

The cuticle in pachyzoids is composed of multiple layers similar to other alcyonidioideans [19, 21], but also shows a reticulate or dendritic pattern on its outermost layer, such as is found in *Haywardozoon* [22], *Pherusella* [21] and *Sundanella* [20]. All of these genera are clearly closely related, but the lack of sequence data for *Haywardozoon* and Pachyzoidae hampers knowing whether this particular cuticular structure is a shared or independently evolved feature.

Rhizoids are, as previously indicated, non-kenozooidal [8] and are hence cystid appendages. They are found in all pachyzoid species on the basal colony side and are essential for anchoring colonies in soft sediment. Based on histological analysis of the current study, specific musculature is present only in rhizoids of *P. grischenkoi* sp. nov.. Its thick cuticle also indicates that movement is likely to be restricted. Possibly hydrostatic pressure increase by polypide retraction could act in movement of the rhizoids. Pachyzoid biology remains virtually unknown, but it is highly unlikely that active colonial movement is possible. Whereas lunuliform cheilostomes have polymorphs with movable bristles [e.g. [23] and conescharellinids have a righting behavior conferred by non-muscular kenozooidal props [24], such structures and behavioral repertoires are lacking in ctenostomes.

## Reproductive aspects of pachyzoids

The current study discovered the presence of some large macrolecithal oocytes in pachyzoids as well as apparent embryo brooded within the zooid, probably the tentacle sheath, in *P. grischenkoi* sp. nov.. These characters indicate that lecithotrophy and brooding probably are a general feature of the family, similar to other ctenostomes [8]. This differs from other deep-sea ctenostomes such as aethozoids [6], the genus *Haywardozoon* [22] and probably the genus *Pierrella* [25], which are zygote-spawners with numerous, smaller oligolecithal oocytes. Deep-sea species of Clavoporidae, however, are also brooders (Schwaha, pers. observation).

Lecithotrophic development and associated coronate larvae are short-lived and hence are generally considered to be correlated with a reduced dispersal rate [see [26–28]. Determination of the numbers of zooids per mature colony revealed that *Jeanloupia zealandica* remains rather small in size, with not more than 11 zooids. In *Pachyzoon*, the number of zooids per colony ranges from c. 20 in *P. atlanticum*, c. 50 in *P. pulvinaris* and almost 100 in *P. grischenkoi*. This implies that species with fewer zooids reach sexual maturity earlier. Since oocytes and embryos appear voluminous, it is possible that larvae result in ancestrulae with multiple zooids, which would confer greater stability on the soft sediments where they live. Although smaller fragmented parts of colonies have been observed in the current study, we have no indication whether fragmentation, as a dispersal form, is present in pachyzoids.

### Distribution of pachyzoids

With the current study, we extend the distribution of Pachyzoidae over a wider geographic range to include New Zealand. The previous report of *P. atlanticum* from New Caledonia pertains to *P. pulvinaris* (see above), which gives it a wider Zealandian distribution in the Southwest Pacific. It seems that pachyzoids have endemic ranges, with *P. atlanticum* occurring exclusively in the North Atlantic and the other three species pertaining to the geological continent of Zealandia, which is mostly submerged [29]. However, likely sampling bias clouds our understanding of the true distributional ranges of pachyzoids, since most samples from the current analyses were from similar areas, mostly southeast of South Island, where all three species may co-occur at the same station (e.g. NIWA Stn S154—see Table 1). There is a high likelihood that pachyzoids may be easily overlooked in other sample analyses or that sampling techniques may be inadequate for capturing them.

So far, all pachyzoid bryozoans are restricted to deeper water ranging from c. 600 m to over 3000 m depth, with most samples occurring around 1000 m depth. As with other deep-sea bryozoans, there is little information on their ecology, including their diet. Given the abundance encountered in the current study, there is a high possibility of finding more samples to discover other aspects of their general biology. The often camouflaged and nondescript appearance of pachyzoids renders them difficult to recognize, especially for non-bryozoologists.

## Conclusions

The analysis of deep-sea samples revealed abundant colonies of pachyzoids belonging to three new species, including one new genus. This shows that the diversity of these bryozoans is much higher than previously known and that they probably constitute an important part of deep-sea ecosystems.

## Data Availability

Data is available on reasonable request.

## Abbreviations

a: Anus
app: Apertural papilla
bbw: Basal body wall
c: Collar
ca: Cardia
cae: Caecum
cy: Cystid appendage
db: Duplicature band
es: Esophagus
fbw: Frontal body wall
kz: Kenozooid
int: Intestine
l: Lophophore
o: Orifice
ooc: Oocytes
oz: Old zooid
ph: Pharynx
plo: Protruded lophophore
pm: Parietal muscles
po: Polypide
pop: Pore-plate
ps: Peristome
psw: Peristomial wall
py: Pylorus
rm: Retractor muscle
te: Testis
ts: Tentacle sheath
v: Vestibulum
vco: Vestibular cone
vw: Vestibular wall
