# Correction: Deep-sea ctenostome bryozoans: revision of the family Pachyzoidae, with description of a new genus and three new species from Zealandia

**DOI:** 10.1186/s40851-024-00229-w

**Published:** 2024-03-11

**Authors:** Thomas Schwaha, Dennis P. Gordon

**Affiliations:** 1 Department of Evolutionary Biology, University of Vienna, Schlachthausgasse 43, 1030 Vienna, Austria; 2 National Institute of Water and Atmospheric Research (NIWA), Wellington, New Zealand

Zoological Letters (2024) 10:4

10.1186/s40851-024-00226-z

Following publication of the original article, it was brought to the journal's attention that due to an error in processing the proofs of the article, the article had published with a duplicate of Figure 6 in place of Figure 5. Figure 5 has since been corrected in the article. The publisher thanks you for reading this erratum and apologizes for any inconvenience caused.

